# Supplementary figures and images for: Selective optogenetic activation of NaV1.7–expressing afferents in NaV1.7-ChR2 mice induces nocifensive behavior without affecting responses to mechanical and thermal stimuli
Source: PLoS One. 2022 Oct 6;17(10):e0275751. doi: 10.1371/journal.pone.0275751 (PMC9536842; doi:10.1371/journal.pone.0275751)

## Slide 1
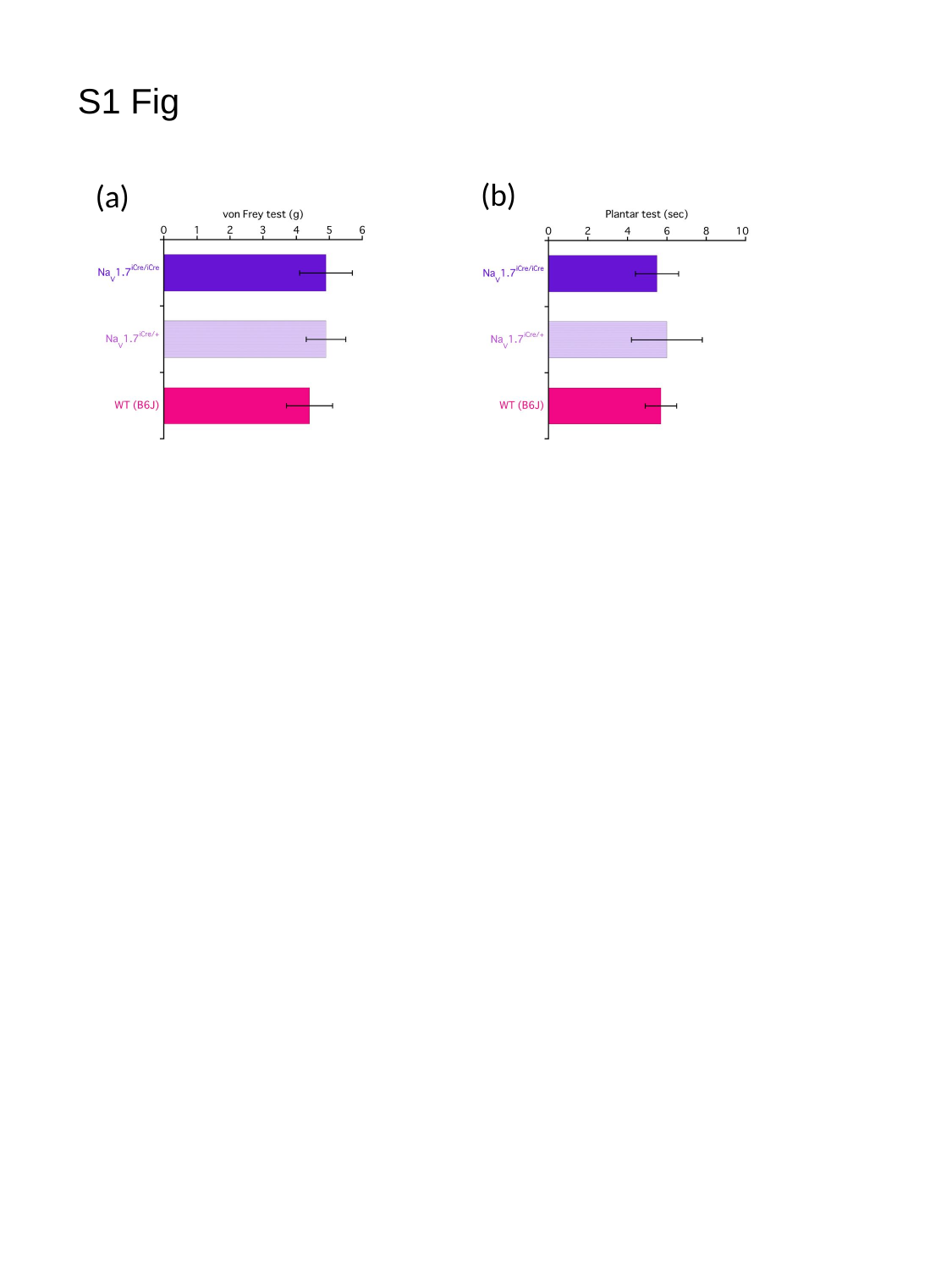

S1 Fig
(b)
(a)

Supplement: S1 Fig — The von Frey test (a) and plantar test (b) were performed with wild-type (WT), NaV1.7iCre/iCre, and NaV1.7iCre/+ mice. The data were analyzed using one-way ANOVA. All results are calculated as mean ± SD of 10 or more animals. Individual results for each strain are (a) WT (B6J): 4.4 ± 0.7 g, NaV1.7iCre/iCre: 4.9 ± 0.8 g, and NaV1.7iCre/+: 4.9 ± 0.6 g, (b) WT (B6J): 5.7 ± 0.8 s, NaV1.7iCre/iCre: 5.5 ± 1.1 s, and NaV1.7iCre/+: 6.0 ± 1.8 s. (PPTX) [file pone.0275751.s001.pptx]

## Slide 1
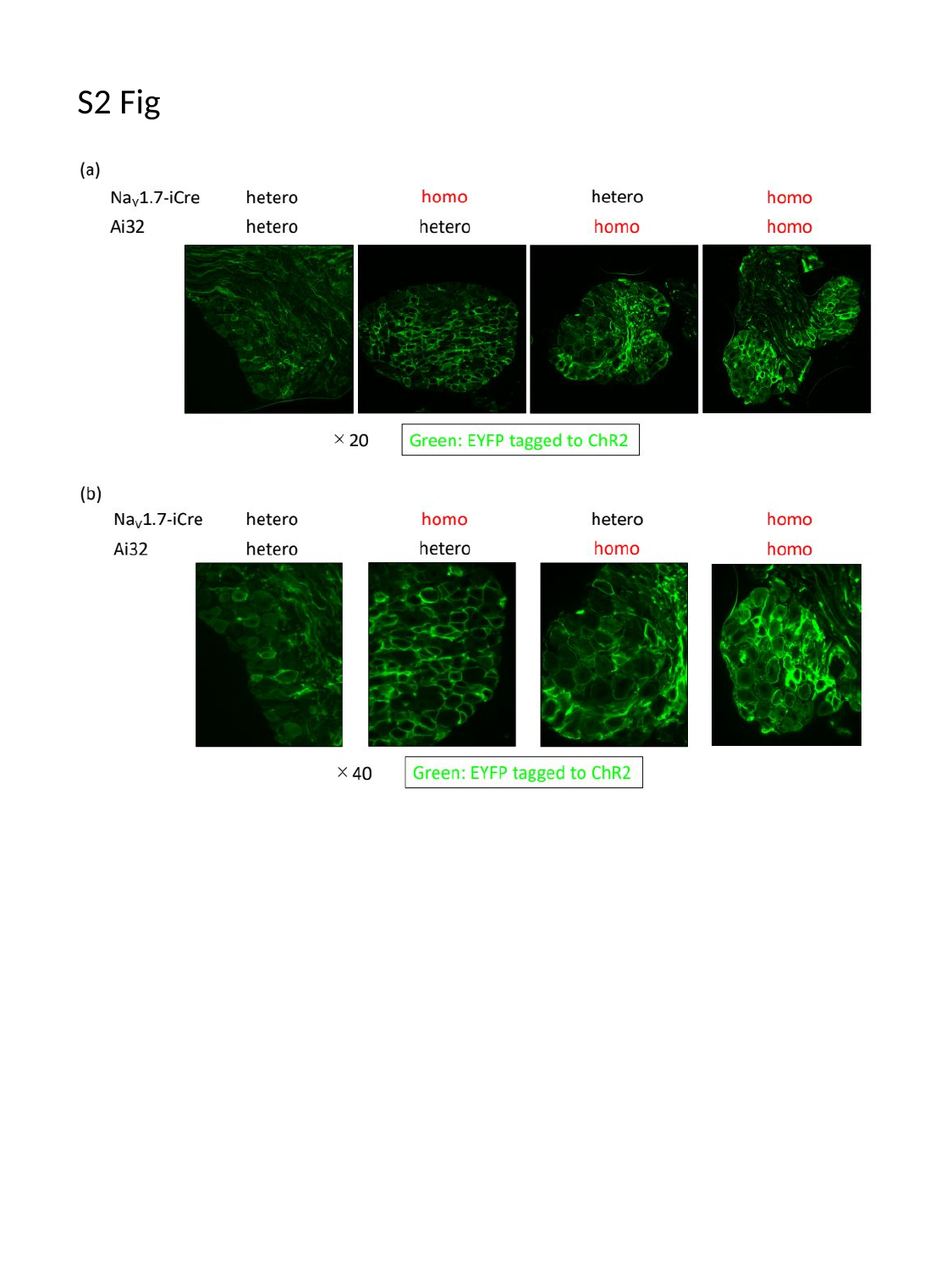

S2 Fig

Supplement: S2 Fig — The green fluorescent signal represents the direct fluorescence of ChR2-EYFP. (a) 20× and (b) 40×. (PPTX) [file pone.0275751.s002.pptx]

# S1 raw images

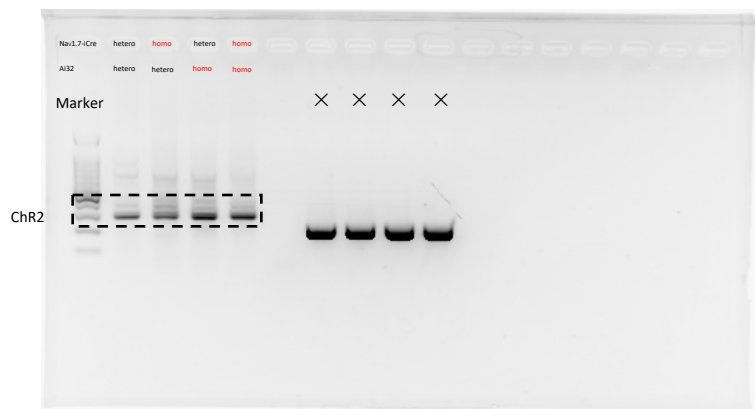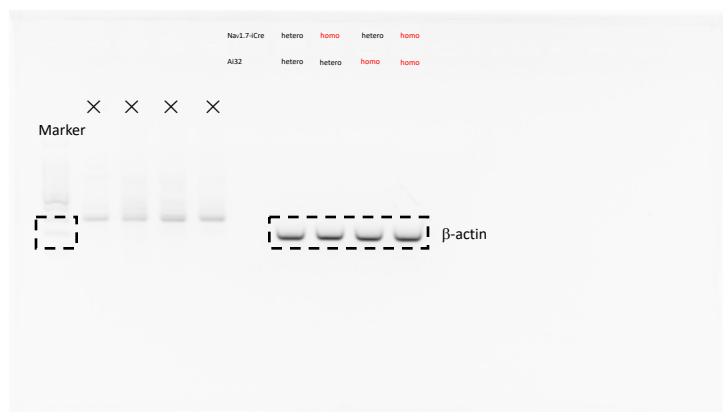

Supplement: S1 Raw images — (PDF) [file pone.0275751.s003.pdf]
