# Supplementary material for: Selective optogenetic activation of NaV1.7–expressing afferents in NaV1.7-ChR2 mice induces nocifensive behavior without affecting responses to mechanical and thermal stimuli
Source: PLoS One. 2022 Oct 6;17(10):e0275751. doi: 10.1371/journal.pone.0275751 (PMC9536842; doi:10.1371/journal.pone.0275751)
Supplement: Attachment — Submitted filename: Response to Reviewers.docx [file pone.0275751.s004.docx]

Dear Dr. Tudor C. Badea,

Thank you for giving me the opportunity to submit a revised draft of my manuscript titled “**Selective optogenetic activation of Na_V_1.7–expressing afferents in Na_V_1.7-ChR2 mice induces nocifensive behavior without affecting responses to mechanical and thermal stimuli”** to PLOS ONE (Manuscript ID: PONE-D-22-16820). We appreciate the time and effort that you and the reviewers have dedicated to providing your valuable feedback on our manuscript. We are grateful to the reviewers for their insightful comments on my paper.

We have extensively revised our paper to reflect most of the suggestions provided by the reviewers. We have highlighted the changes within the manuscript in red font. Here is a point-by-point response to the reviewers’ comments and concerns.

Reviewer #1

1) the manuscript title is misleading (or wrong). The mechanical and thermal tests (no deficits) have nothing to do with ontogenetic activation of Na_V_1.7-Cre+ neurons. These are baseline behaviors. Thus, the title must be changed.

Response: The transgenic mice used in our study have a knock-in of the iCre recombinase gene downstream of *Scn9a* in the Na_V_1.7 gene. Therefore, we were concerned about the normal expression and functioning of Na_V_1.7. As mentioned in the discussion section (Page 24, lines 263-270), Na_V_1.8 currents were not observed in previously generated Na_V_1.8-Cre homozygote mice. Therefore, the nociceptive response to mechanical and thermal stimuli was likely attenuated. To address this concern, we wanted to demonstrate that the nociceptive response to mechanical and thermal stimuli, which involves functioning of the Na_V_1.7, is at least normal. Therefore, we included the phrase “without affecting responses to mechanical and thermal stimuli” in the manuscript title.

2) There is little data showing characterizing of the new Na_V_1.7-Cre line. The illustration showing the genomic structure, but no PCR or sequencing validating insert as anticipated. There is no co-staining of reporter (GFP) with endogenous Na_V_1.7 (or double in situ) or other common DRG neuron markers, CGRP, IB4, NF200. For the DRG section images, only GFP, and there is no quantification!!!

Response: We omitted the quantification of ChR2 in the immunostaining of DRG because we used PCR to assess the expression of ChR2 in the DRG of each phenotype. Immunostaining images of “Distribution of ChR2-EYFP channels in DRG” for mice of the four genotypes has been provided as supplementary information (S2 Fig). In addition, we added a figure showing Na_V_1.7 and ChR2-EYFP in the DRG of Na_V_1.7^iCre/+^;Ai32/+ mouse (Fig 2b). We revised the text in the manuscript accordingly (Page 14: lines 147-151).

“Fig 2b shows a typical immunohistochemical image showing Na_V_1.7 and ChR2-EYFP in the DRG of Na_V_1.7^iCre/+^;Ai32/+ mouse. EYFP fluorescence was observed in Na_V_1.7-expressing DRG neurons. EYFP fluorescence was also observed in the dorsal horn of the spinal cord and was faded in the deep and superficial layers of the glabrous skin bordering the dermal– epidermal junction.”

Although staining of common DRG neuron markers was not performed in this study, we intend to perform such experimentation in the future.

3) For peripheral light triggered paw withdrawal reflex, how do the authors know that it indicates pain response? Fos immunostaining with spinal cord sections after blue light stimuli would help to strengthen this point.

Response: We assessed nocifensive behavior and did not focus on proving whether the mice strictly felt pain. We believe that the transgenic mice developed in this study is a mouse model for pain; however, we have differentiated between “nocifensive” and “painful” as much as possible.

Examining the changes in Fos expression induced by short-term light irradiation would be difficult. For instance, Daou et al. demonstrated the ipsilateral c-Fos expression in neurons of laminae I–III of the dorsal horn after 10 min suprathreshold blue-light stimulation of the left hindpaw of *Na_V_1.8 – ChR2^+^* mice (J. Neurosci. 2013; 33: 18631–18640). Currently, we are investigating the persistence of nociceptive behavior after prolonged light exposure and considering the examination of changes in pain-related molecule expressions in the spinal cord and DRG.

4) Other suggestion is to combine figures. Each current figure contains only one panel.

Response: The number of figures has been reduced to six in total, because the old Fig. 1 and 2 were combined into new Fig. 3 and the old Fig. 6 was changed to S2 Fig.

5) In addition to the experiments/data, the writing is also problematic. The description of generating Na_V_1.7 mouse line should be a main result section (the first part), which was put in the method instead. The results part overall is too short and over simplified. On the other hand, the discussion is lengthy and some parts (like Mrgd-ChR2 mice) are even irrelevant. The entire manuscript will need to be re-written.

Response: The sections "Development of original Na_V_1.7–iCre (Na_V_1.7^iCre/+^) knock-in mice" and "Generation of the four genotypes of Na_V_1.7-ChR2 mice" were moved to the Results section (Pages 9-12, lines 92-128) from the Methods section.

To address the issue of a lengthy discussion, the discussion regarding Mrgprd-ChR2 and TRPV1-ChR2 mice in the discussion section has been shortened (Pages 22-23, lines 243-250).

Reviewer #2

1) As a general observation, the Results section is extremely succinct, and the overall description of the data lacks quantitative detail. A more in-depth presentation of the actual results is warranted. For example, the authors limit their description of the nocifensive results is warranted. For example, the authors limit their description of the nocifensive behaviour elicited by blue light to paw withdrawal (in Fig.3) and they do not discuss other manifestations which are generally associated with pain behaviour (paw licking, jumping, vocalization, etc...). Moreover, an important control is missing from the data shown in Fig.3: the experimenters should have used light of a different frequency (yellow light, at 590 nm, for example) at the highest light intensity used in the experiment, to rule out a ChR-independent effect of strong illumination on the animal behaviour. Finally, the light intensity used in the experiments illustrated in Fig.3 should be provided in mW/mm2, not in mW.

Response: We did not assess other nociceptive responses separately in detail in this study. We did not observe jumping and vocalization by mice, which are generally associated with nocifensive behavior, during experimentation. Paw licking was occasionally observed and was considered a positive nocifensive behavior, as was paw withdrawal. Therefore, the method text was revised as follows.

Page 32, Lines 354-355: “The percentage of trials during which hind paw withdrawal or paw licking occurred was recorded.”

Indeed, this comment by reviewer #2 made us realize the importance of eliminating the ChR2-independent effects of strong illumination on animal behavior. Unfortunately, phenotypes other than Na_V_1.7^iCre/+^;Ai32/+ mice are not maintained due to breeding space issues. Consequently, an extended amount of time would be required to obtain a suitable number of these mice phenotypes other than Na_V_1.7^iCre/+^;Ai32/+ for this control experiment. Hence, as a control experiment, we exposed the Na_V_1.7^iCre/+^;Ai32/+ mice to yellow light. This was reflected in the manuscript by adding the following sentences in the Results section.

Pages 17-18, Lines 191-195: “To rule out a ChR2-independent effect of strong illumination on the animal behavior, we performed a yellow LED light irradiation hind paw withdrawal test on NaV1.7iCre/+;Ai32/+ mice. Irradiation with yellow light irradiation (5 mW) did not induce hind paw withdrawal behavior.”.

The light power meter that we used (LPM-100™; Bioresearch Center Inc., Aichi, Japan) displays light intensity in units of “mW”; to convert it to “mW/mm^2^”, we divided the light intensity by the area of illumination (48 mm^2^). Accordingly, the added the following sections to the methods section.

Page 31, Lines 351-353: “Since the power meter measures light intensity in milliwatts (mW), the light density in mW/mm^2^ was calculated by dividing the light intensity by the illuminated area in square millimeters (48 mm^2^).”

2) There is no attempt to quantify and characterise in more detail the expression of ChR-EYFP in DRG cell bodies in the sections illustrated in Fig.6. More information should be provided in terms of what percentage of neurons present YFP fluorescence, what is their average size, etc... It would also be of interest to dissect the expression of ChR-EYFP in peptidergic versus non-peptidergic neurons, using an anti-CGRP and/or an anti-P2X3 antibody, for instance.

Response: We omitted the quantification of ChR2 in the immunostaining of DRG because we used PCR to assess the expression of ChR2 in DRG of each phenotype. Immunostaining images of “Distribution of ChR2-EYFP channels in DRG” for mice of the four genotypes has been provided as supplementary information (S2 Fig). As reviewer #2 pointed out, it would be interesting to characterize ChR2 expression using anti-CGRP and anti-P2X3 antibodies. Currently, we are developing mice targeting Na_V_1.8 and Na_V_1.9 as well as Na_V_1.7-ChR2 mice and will report on this subject in our next study.

3) It would be of interest to investigate and confirm the trafficking of ChR to peripheral and central endings of Na_V_1.7-expressing peripheral nociceptors, by monitoring YFP fluorescence in skin and spinal cord sections.

Response: We monitored the EYFP fluorescence in the dorsal horn spinal cord and hindpaw glabrous skin (Fig. 2b). Page 14, lines 149-151: “EYFP fluorescence was also observed in the dorsal horn of the spinal cord and was faded in the deep and superficial layers of the glabrous skin bordering the dermal– epidermal junction.”
